# Characterizing executive functioning and associated behaviors in individuals with dual-specificity tyrosine phosphorylation-regulated kinase 1A (*DYRK1A*) syndrome

**DOI:** 10.3389/fnins.2024.1485499

**Published:** 2025-01-07

**Authors:** Hannah M. Rea, Sara Jane Webb, Evangeline C. Kurtz-Nelson, Caitlin M. Hudac, Raphael A. Bernier, Conor Miles, Rachel Earl, Alana Whiting, Curtis Eayrs, Margaret Johansson, Tianyun Wang, Evan E. Eichler, Emily Neuhaus

**Affiliations:** ^1^Department of Psychiatry and Behavioral Sciences, University of Washington School of Medicine, Seattle, WA, United States; ^2^Seattle Children’s Research Institute, Center on Child Health, Behavior and Development, Seattle, WA, United States; ^3^Department of Pediatrics, Indiana University School of Medicine, Indianapolis, IN, United States; ^4^Department of Psychology, University of South Carolina, Columbia, SC, United States; ^5^Carolina Autism and Neurodevelopment Research Center, University of South Carolina, Columbia, SC, United States; ^6^Department of Medical Genetics, Center for Medical Genetics, School of Basic Medical Sciences, Peking University, Beijing, China; ^7^Neuroscience Research Institute, Peking University, Ministry of Education of China & National Health Commission of China, Beijing, China; ^8^Autism Research Center, Peking University Health Science Center, Beijing, China; ^9^Department of Genome Sciences, University of Washington School of Medicine, Seattle, WA, United States; ^10^Howard Hughes Medical Institute, University of Washington, Seattle, WA, United States

**Keywords:** *DYRK1A*, executive functioning, ADHD, autism, cognitive functioning

## Abstract

**Introduction:**

*DYRK1A*, a protein kinase located on human chromosome 21, plays a role in postembryonic neuronal development and degeneration. Alterations to *DYRK1A* have been consistently associated with cognitive functioning and neurodevelopmental disorders (e.g., autism, intellectual disability). However, the broader cognitive and behavioral phenotype of *DYRK1A* syndrome requires further characterization. Specifically, executive functioning, or cognitive processes that are necessary for goal-directed behavior, has not yet been characterized in this population.

**Methods:**

Individuals with *DYRK1A* variants (*n* = 29; ages 4 to 21 years) were assessed with a standardized protocol with multiple measures of executive functioning: Delis-Kaplan Executive Function Schedule, and chronologically age-appropriate caregiver-report forms of the Behavior Rating Inventory of Executive Function (BRIEF) and Achenbach System of Empirically Based Assessment (ASEBA). We first examined the feasibility and appropriateness of established executive functioning measures among participants with *DYRK1A* syndrome to inform selection of executive functioning tools in future research. We then characterized executive functioning among the group, including associations with other phenotypic features.

**Results:**

Neurocognitive assessments of executive functioning were deemed infeasible due to cognitive and verbal functioning. Caregiver-report revealed elevated executive functioning concerns related to self-monitoring, working memory, and planning/organization on the BRIEF, and attention and ADHD on the CBCL. Only two participants had existing ADHD diagnoses; however, 5 participants (out of 10 participants with data) exceeded the cutoff on the BRIEF, 13 individuals (out of 27 with data) exceeded the cutoff on the ASEBA ADHD subscale, and 18 exceeded the cutoff on the ASEBA attention subscale. There was concordance between ADHD diagnosis and the ASEBA, but not BRIEF. Executive functioning was correlated with nonverbal IQ and autism traits.

**Discussion:**

Objective measures of executive functioning are needed for individuals with intellectual disability who are nonverbal and/or have motor limitations. Diagnostic overshadowing, or the tendency to attribute all problems to intellectual disability and to leave other co-existing conditions, such as executive functioning challenges or ADHD, undiagnosed, is common. Phenotypic characterization of executive functioning is therefore important for our understanding of *DYRK1A* syndrome and for ensuring that caregivers’ concerns are addressed, and individuals receive the clinical services that best meet their needs.

## 1 Introduction

*DYRK1A* (dual-specificity tyrosine phosphorylation regulated kinase 1A), a protein kinase located in the Down syndrome critical region of human chromosome 21, plays a major role in early neural commitment, precursor proliferation, neurogenesis, as well as in postembryonic neuronal development and degeneration, and, therefore, cognitive functioning early in life and across the lifespan (Wegiel et al., 2011; Cortes et al., 2024). *DYRK1A* syndrome is characterized by haploinsufficiency, or under expression of *DYRK1A*, as well as developmental delay and intellectual disability, speech problems, microcephaly, and other neurodevelopmental disorders such as autism spectrum disorder (ASD; Van Bon et al., 2016; Fenster et al., 2022; Kurtz-Nelson et al., 2023). Conversely, overexpression of *DYRK1A* also confers neurodevelopmental risk, as it is believed to contribute to neurocognitive deficits associated with Down syndrome (Park and Chung, 2013). This complex role of *DYRK1A* in neurodevelopmental functioning necessitates in-depth, standardized characterization of the range of cognitive and behavioral presentations associated with *DYRK1A* syndrome.

Executive functioning, higher order cognitive processes that strongly impact behavioral functioning, has been shown to be an area of challenge among neurogenetic and neurodevelopmental disorders that are associated with *DYRK1A* (Demetriou et al., 2019; Tungate and Conners, 2021). Executive functioning is an umbrella term for cognitive processes that underlie goal-directed behavior, including skills such as planning, organization, working memory, inhibition, and cognitive flexibility (Jurado and Rosselli, 2007). Among individuals with neurodevelopmental conditions, executive functioning skills and challenges vary substantially, depending both on the subdomains measured and the assessment type (Demetriou et al., 2018). There is limited knowledge of the executive functioning profile within *DYRK1A* syndrome, although executive functioning challenges are likely prevalent among those with *DYRK1A* syndrome due to their association with other, related neurodevelopmental disorders. For example, on average, autistic individuals show a global impairment in executive functioning compared to non-autistic individuals (Demetriou et al., 2018). Similarly, those with intellectual disability exhibit more challenges with executive functioning compared to mental-age matched peers (Spaniol and Danielsson, 2022). Moreover, executive functioning may be an important link between IQ and adaptive functioning, or daily living, skills (Fidler and Lanfranchi, 2022; Onnivello et al., 2022).

As executive functioning is a broad term, there are numerous ways to operationalize and assess this multi-faceted construct (Duggan and Garcia-Barrera, 2015). When feasible and available, standardized neurocognitive assessments administered by trained examiners can be used to assess executive functioning. Commonly used neurocognitive assessments of executive functioning are the Delis-Kaplan Executive Function System (D-KEFS; Delis et al., 2001) Verbal and Design Fluency tasks, which measure fluency and cognitive flexibility, and the Color Word Interference Task, which measures inhibition. Both have been utilized among individuals with intellectual disability, have been shown to have convergent and discriminant validity for individuals with intellectual disability, and were sensitive measures of executive functioning in youth with intellectual disability (Erostarbe-Pérez et al., 2022). Because neurocognitive tests rely on examiner administration rather than caregiver report, these tools are sometimes considered to be more objective assessments than other options (Toplak et al., 2013). However, valid administration may require motoric behaviors and/or communication that reduce their usefulness among populations with neurodevelopmental conditions, such as *DYRK1A* syndrome, with known behavioral and hypotonia issues (Fenster et al., 2022). Other questions surrounding validity and feasibility may arise as the minimum age for many executive functioning neurocognitive measures is school-age (e.g., 7 to 8 years depending on the D-KEFs subtest; Delis et al., 2001). Due to the high rates of intellectual disability among those with *DYRK1A* syndrome, the administration of the tasks as well as the chronological-age standardization for scoring may be above the developmental level of those with *DYRK1A* syndrome (van Bon et al., 2011).

Caregiver reports of executive functioning, in the form of normed and standardized questionnaires, are most often used in research and clinical settings, as they are relatively cost effective and time efficient in administration and scoring, and correlate with daily functioning (Gardiner et al., 2017; Ten Eycke and Dewey, 2016). Caregiver report also allows for evaluation of executive functioning among a heterogenous and inclusive population of individuals with a wide range of ages, without constraints related to attention, communication, sensory-motor needs, or age (Fidler and Lanfranchi, 2022; Ten Eycke and Dewey, 2016). A commonly used caregiver-report measure of executive functioning is the Behavior Rating Inventory of Executive Function (BRIEF; Gioia et al., 2000). Although originally developed with neurotypical populations, the BRIEF is increasingly administered among populations with neurodevelopmental and neurogenetic conditions, including Down syndrome and autism, as it is sensitive to executive functioning challenges in youth with intellectual disability (Fidler and Lanfranchi, 2022; Hendrickson and McCrimmon, 2019; Lee et al., 2011).

Broad-based, standardized caregiver-report measures of emotional and behavioral problems often also capture executive functioning. For example, the Achenbach System of Empirically Based Assessment (ASEBA) includes Attention and ADHD subscales (Achenbach and Rescorla, 2006), which include items capturing impulsivity and the ability to monitor and inhibit behavior. Among the general population, ASEBA caregiver-reports are commonly used as they are reliable, valid, easy-to-use assessments that capture a range of concerns (Biederman et al., 2021). Newer research also indicates that these are reliable and valid for use with children with intellectual disability, such as children with Down syndrome (Esbensen et al., 2018). For example, on the preschool (ages 1.5 to 5 years) and school-age (ages 6 to 18 years) Child Behavior Checklist versions of ASEBA, the most common problems reported by caregivers of youth with *DYRK1A* syndrome were attention problems (Fenster et al., 2022). However, again, the discrepancy between chronological and mental age for those with *DYRK1A* syndrome means that the administration form selected, and age-based standardization may impact the validity of these caregiver reports.

To better understand the cognitive and behavioral phenotype associated with *DYRK1A*, the following study aimed to present a clinical characterization of executive functioning in individuals with *DYRK1A* syndrome. We first examine the feasibility and appropriateness of established executive functioning measures among participants with *DYRK1A* syndrome to inform selection of executive functioning tools in future research. We then characterize executive functioning among the group, with attention to associations between executive functioning and other phenotypic features (autism and ADHD). We compared executive functioning to autism and ADHD as both neurodevelopmental disorders are highly correlated with challenges in executive functioning, and ADHD is typically diagnosed using measures of executive functioning among the general population (Peterson et al., 2024).

## 2 Methods

### 2.1 Procedure

Participants were enrolled as part of genetics-first research projects (R01MH101221) at the University of Washington (UW), aimed at in-depth phenotyping of cognitive functioning (including executive functioning), autism traits, and mental health of individuals with disruptive variants in a variety of genes associated with ASD (Kurtz-Nelson et al., 2023; Beighley et al., 2020). Following informed consent, and, when appropriate, assent, families participated in-person at UW, in their home via clinician visit, or remotely via telehealth during the COVID-19 pandemic. Although precise assessment protocols varied slightly based on visit type/location, a consistent set of domains were assessed (e.g., cognition, communication, ASD, mental health) through standardized caregiver-report measures and behavioral evaluations administered by clinicians naïve to gene group membership. All assessments were administered by or under the supervision of clinical psychologists.

### 2.2 Participants

Participants in the current paper included 29 individuals with disruptive, pathogenic single nucleotide variants (nonsense, splice site, frameshift, or missense mutations) at the *DYRK1A* gene. See Supplementary materials for full variant information. Participants ranged in chronological age from 4 years 0 months to 21 years 10 months (*M* = 9 years 9 months, *SD* = 5 years, 9 months). Both females (*n* = 13) and males (*n* = 16) were included. See Table 1 for sample characteristics.

**Table 1:** Demographics and descriptive statistics.

Domain	M(SD)	Range	%(n)
Chronological age	9 years 9 months (5 years 9 months)	4 years to 21 years 10 months	–
FSIQ standard score	44.54 (25.96)	10 to 133	3.4% (1)
VIQ standard score	42.93 (27.04)	4 to 119	6.9% (2)
NVIQ standard score	45.63 (26.11)	12 to 133	6.9% (2)
Domain	%(n)	–	Missing %(n)
Female %(n)	44.8% (13)	–	0% (0)
ASD diagnosis %(n)	82.8% (24)	–	0% (0)
ID diagnosis	89.7% (26)	–	0% (0)

FSIQ, Full scale IQ; VIQ, Verbal IQ; NVIQ, Nonverbal IQ; ASD, Autism spectrum disorder; ID, Intellectual disability.

### 2.3 Measures

#### 2.3.1 Executive functioning

##### 2.3.1.1 Neurocognitive tests

The Delis-Kaplan Executive Function System (D-KEFS) Verbal Fluency, Design Fluency, and Color-Word Interference subtests were included and have validated age ranges of 8 to 79 years (Delis et al., 2001). The Verbal Fluency Test measures an individual’s letter fluency, category fluency, and category switching. The Design Fluency Test evaluates a person’s ability to simultaneously adhere to task rules and restrictions while generating visual patterns and their ability to switch between task rules. The Color-Word Interference task assesses cognitive flexibility and verbal processing speed.

##### 2.3.1.2 Caregiver/informant report

Caregiver/other-informant reports included the Behavior Rating Inventory of Executive Function (BRIEF; Gioia et al., 2000; Gioia and Isquith, 2011) and the Achenbach System of Empirically Based Assessment (ASEBA; Achenbach and Rescorla, 2006; Rescorla, 2005). For each participant, the administered version was matched to the individual’s chronological age, with a breakdown of form administration based on chronological age and compared to mental age shown in Table 2.

**Table 2:** Caregiver/self-report versions of executive functioning measures administered.

	Preschool % (n)	School-age % (n)	Adult % (n)	Missing % (n)	Match to mental age % (n)	Total completed (n)
BRIEF	40% (4)	50% (5)	10% (1)	64.3% (18)	30% (3)	(10)
ASEBA	44.4% (12)	51.9% (14)	3.7% (1)	7.4% (2)	51.9% (14)	(27)

The BRIEF includes a pre-school version (2 years – 5 years 11 months), a school-age version (5–18 years), and an adult informant report (18+ years). All versions yielded T-scores for several subscales and domain scores, with the Global Executive Composite (GEC) representing overall executive functioning. Subscales and domains scores are listed in Table 3. T-scores below 65 are considered in the normal range and T-scores 65 or higher indicate clinically significant concerns. The BRIEF also includes two validity scales. The Inconsistency validity scale indicates the extent to which similar items were endorsed in inconsistent manners, with a higher score indicating more inconsistent responses, and scores of 8 or higher indicating inconsistent responses. The Negativity validity scale measures a tendency for the respondent to answer in an unusually negative manner relative to a clinical sample, with higher scores indicating more negativity. On the preschool form scores of 4 or higher on the Negativity scale are considered elevated, on the school-age form scores of 5 or higher are considered elevated, and on the adult form scores of 6 or higher are considered elevated.

**Table 3:** Descriptive statistics of caregiver/self-report of executive functioning.

Measure	M (SD)	Range	%(n) Above clinical cut-off
BRIEF subscale T-scores			
Inhibition (*n* = 10)	60.60 (14.47)	39 to 88	30.0% (3)
Shift (*n* = 10)	55.30 (10.35)	38 to 68	20.0% (2)
Emotional control (*n* = 10)	46.90 (12.64)	36 to 71	10.0% (1)
Self-monitoring (*n* = 6)	65.17 (17.86)	40 to 91	66.7% (4)
Initiation (*n* = 6)	60.83 (18.34)	36 to 91	33.3% (2)
Working memory (*n* = 10)	69.70 (17.83)	39 to 98	70.0% (7)
Planning/organization (*n* = 10)	65.70 (15.37)	38 to 90	50.0% (5)
Organization of materials (*n* = 6)	53.5 (12.85)	39 to 66	33.3% (2)
BRIEF domain scores			
Behavioral regulation index (*n* = 6)	54.00 (12.52)	38 to 73	16.7% (1)
Metacognition index (*n* = 10)	69.90 (19.19)	36 to 99	60.0% (6)
Global executive composite (*n* = 10)	64.30 (14.38)	36 to 86	50.0% (5)
ASEBA			
ADHD scale (*n* = 27)	62.37 (8.11)	50 to 76	48.1% (13)
Attention Scale (*n* = 27)	69.74 (10.62)	52 to 93	66.7% (18)

Evaluating behavioral manifestation of executive function, the ASEBA is a collection of questionnaires created to screen common emotional and behavioral problems in community settings. It contains a series of versions across ages: the Child Behavior Checklist (CBCL) Preschool Form (1.5–5 years old), the CBCL for school age children (6–18 years old), and Adult Informant-Report (ABCL; 18+ years old). All versions include subscales that assess “Attention Problems” (symptom domain) and “Attention Deficit Hyperactivity Disorder” (ADHD; DSM-oriented subscale) using T-scores with a mean of 50 and standard deviation of 10. T-scores below 65 (i.e., below the 95th percentile) are considered in the normal range (Achenbach and Rescorla, 2006; Mulraney et al., 2022), and T-scores 65 or higher indicate borderline or clinically significant concerns.

#### 2.3.2 Cognition

Full scale IQ, Non-Verbal IQ, and Verbal IQ were derived from mental age-appropriate standardized measures. IQ scores were generated using standardized deviation scores (*M* = 100, *SD* = 15) or ratio scores (mental age equivalent / chronological age x 100) if the participant’s performance was below the floor of the measure and could not be calculated as a deviation score. Mental age equivalent scores were also generated.

Three measures of intellectual ability were used based on clinician judgment and the mental age of each individual: Differential Ability Scales, 2^nd^ Edition (DAS-II; Elliot, 2007; *n* = 16), or the Wechsler Abbreviated Scales of Intelligence, 2^nd^ Edition (WASI-II; Wechsler, 2011; *n* = 4). The Mullen Scales of Early Learning (Mullen, 1995; *n* = 8) was used for participants who were seen in-person, were unable to complete DAS-II or WISC-IV items, and whose mental age was below 4 years, per caregiver report measures and expert clinician judgment. The Developmental Profile (DP-4; Alpern, 2020; *n* = 1) cognitive scale standard score was used to obtain a Full Scale IQ for participants who were seen remotely during the COVID-19 pandemic and were unable to complete WASI-II items. Cognitive test selection and procedures were derived from the Simon Simplex Collection (Fischbach and Lord, 2010).

#### 2.3.3 Neurodevelopmental diagnoses

Neurodevelopmental disorder diagnoses were assessed in two ways. ADHD was ascertained based on caregiver report of a past or current diagnosis from a medical/psychological professional during a medical history interview. Autism diagnoses were attained through clinical best estimate of the research team following each participant’s study completion using all available information, which included the Autism Diagnostic Interview-Revised (ADI-R; Lord et al., 1994), Autism Diagnostic Observation Schedule, Second Edition (ADOS-2; Lord et al., 2008), and/or the Social Responsiveness Scale, Second Edition (SRS-2; Constantino, 2012).

#### 2.3.4 Autism traits

The Social Responsiveness Scale, Second Edition (SRS-2) is a 65-item caregiver report of autism behaviors (Constantino, 2012). On the SRS-2, parents report their child’s behavior over the past 6 months on a 4-point Likert scale (1 = “not true,” 4 = “almost always true”). The SRS-2 includes five subscales: social awareness, social cognition, social communication, social motivation, and RRBs. The first four subscales may also be summed into a composite score called the Social Communication Index. All 5 subscales may be summed into a total composite score. T-scores are generated for total and subscale scores with a mean of 50 and standard deviation of 10, with higher scores indicating higher levels of behaviors associated with ASD.

### 2.4 Analytic plan

To assess feasibility and appropriateness of measures, we reviewed the number of administrations completed, reason for non-completion, and the match between the chronological and mental age for caregiver-report questionnaires. To characterize executive functioning, we summarized the descriptive statistics of subscale and total scores on executive functioning measures and calculated the percentage of the sample with clinically significant scores (T-score 65 or higher). To consider associations between executive functioning and broader phenotypic features, we conducted Spearman’s correlations between executive functioning with age (chronological and mental), IQ (full-scale, verbal, and nonverbal) and autism traits. To further characterize the association between executive functioning and cognitive functioning, we also used *t-*tests to compare those above v. below the clinically significant cut-point on executive functioning measures on verbal IQ (VIQ) as well as on nonverbal IQ (NVIQ). We focused on VIQ and NVIQ, rather than FSIQ, given differences between VIQ and NVIQ for individuals with *DYRK1A* syndrome. We then investigated correspondence between clinical significance on executive functioning measures and ADHD diagnoses by presenting sample sizes for those with/without clinically significant concerns on executive functioning measures compared to those with/without an ADHD diagnosis.

## 3 Results

### 3.1 Feasibility

**Figure 1:**
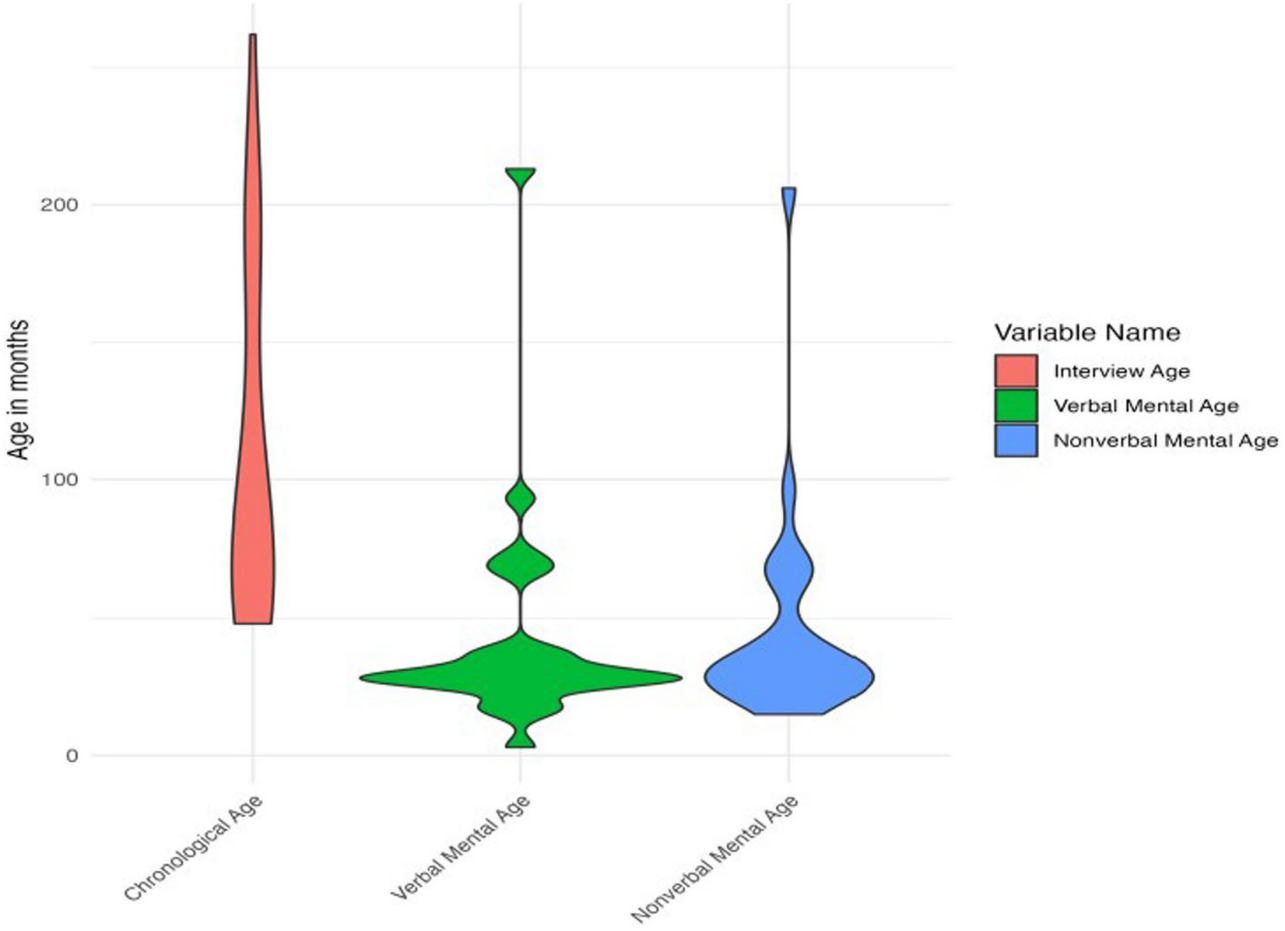
Violin plot of chronological and mental ages.

Participants’ chronological ages ranged from 4 years to 21 years 10 months (48 to 262 months). IQ data were available for 28 participants. Verbal and nonverbal mental ages could not be computed for the 4 participants who completed the WASI-II for IQ testing and for the one participant who did not have IQ data. For the 24 participants with available data, verbal mental ages ranged from 3 months to 17 years 9 months (3 to 213 months) and nonverbal mental ages ranged from 1 year 3 months to 17 years 2 months (15 to 206 months) at the time of participation. See Figure 1 for plots of chronological and mental ages.

The D-KEFS and BRIEF were removed from the study protocol over time, in part to streamline the assessment battery and reduce burden on families. The D-KEFS was also removed due to low rates of task completion. The D-KEFS was part of the assessment battery for 9 participants, but only 4 of these participants had a chronological age above the minimum age for administration (>8 years). The Verbal Fluency and Color-Word Interference tasks were validly completed by 2 of those 4 participants (chronological ages 10 years 3 months and 12 years 11 months), both of whom had mental ages greater than 5 years. The D-KEFS was attempted with one other participant (chronological age 19 years 10 months, mental age not available) but the participant declined. The other participant with a chronological age in the appropriate age range was nonverbal and therefore could not complete the tasks. The Design Fluency Task on the D-KEFS was attempted by 4 participants and discontinued when participants did not understand the directions. The D-KEFS Design Fluency Task was not administered to the other 5 participants as they were out of age range. Of the 3 participants who completed the D-KEFs, scores were either Well Below Average (scaled score = 1–2) or Below Average (scaled score = 7) on Letter Fluency, indicating a range of ability to generate words that start with a certain letter while following rules and restrictions. On Category Fluency, which requires participants to quickly say words that fit in a specified category, as well as Category Switching, which asks participants to say words that alternate between two categories, scores were Well Below Average (Scaled Score = 1–4). Again, no participants completed the Design Fluency task, so data were not available.

BRIEF data were available for 10 participants and ASEBA data were available for 27 participants. Most participants with completed BRIEF and/or ASEBA data received an assessment of executive functioning that matched their chronological age but was higher than their mental age. See Table 2 for a distribution of BRIEF and ASEBA versions that were administered, relative to both chronological age and computed mental age.

Again, the high rates of missing data on the BRIEF were due to changes to the study protocol. About 75% of males were missing BRIEF data (*n* = 12), and 53.8% of females were missing BRIEF data (*n* = 7). Based on Wilcoxon t-test, used to account for the small sample size and non-normal distribution of the data, there was not a significant difference in chronological age (W = 109.5, *p* = 0.521) or mental age (verbal: W = 51, *p* = 0.327; nonverbal: W = 44, *p* = 0.168) between those who did or did not have BRIEF data. Scores on the Inconsistency validity scale ranged from 0 to 7 (*M* = 3.6, *SD* = 2.22) but all forms were considered acceptable. Scores on the Negativity validity scale ranged from 0 to 5 (*M* = 2, *SD* = 1.89). Scores were considered elevated on the Negativity scale for 2 participants who received the pre-school version of the measure. These participants’ scores were retained in analyses as they were still considered valid based on other validity scales (i.e., Inconsistency scores and clinician rating of validity). Both participants who had missing data on the ASEBA were male and had mental ages of 28 months. However, their chronological ages were quite different (6 years 10 months and 21 years 10 months).

### 3.2 Executive functioning—informant report

**Figure 2:**
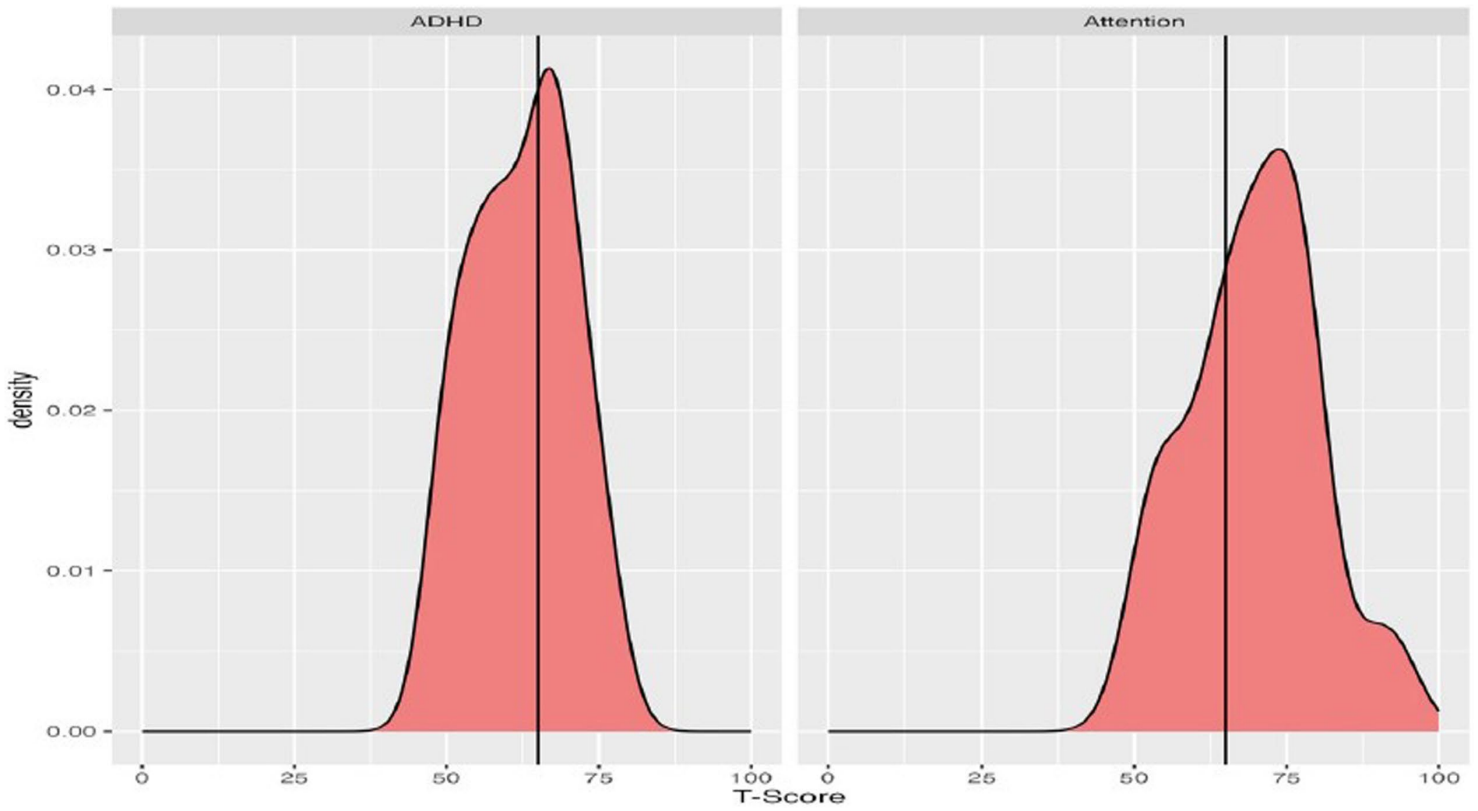
Density plots of T-scores on the BRIEF, with a vertical line demarcating clinically significant scores (T-scores 65 or higher).

**Figure 3:**
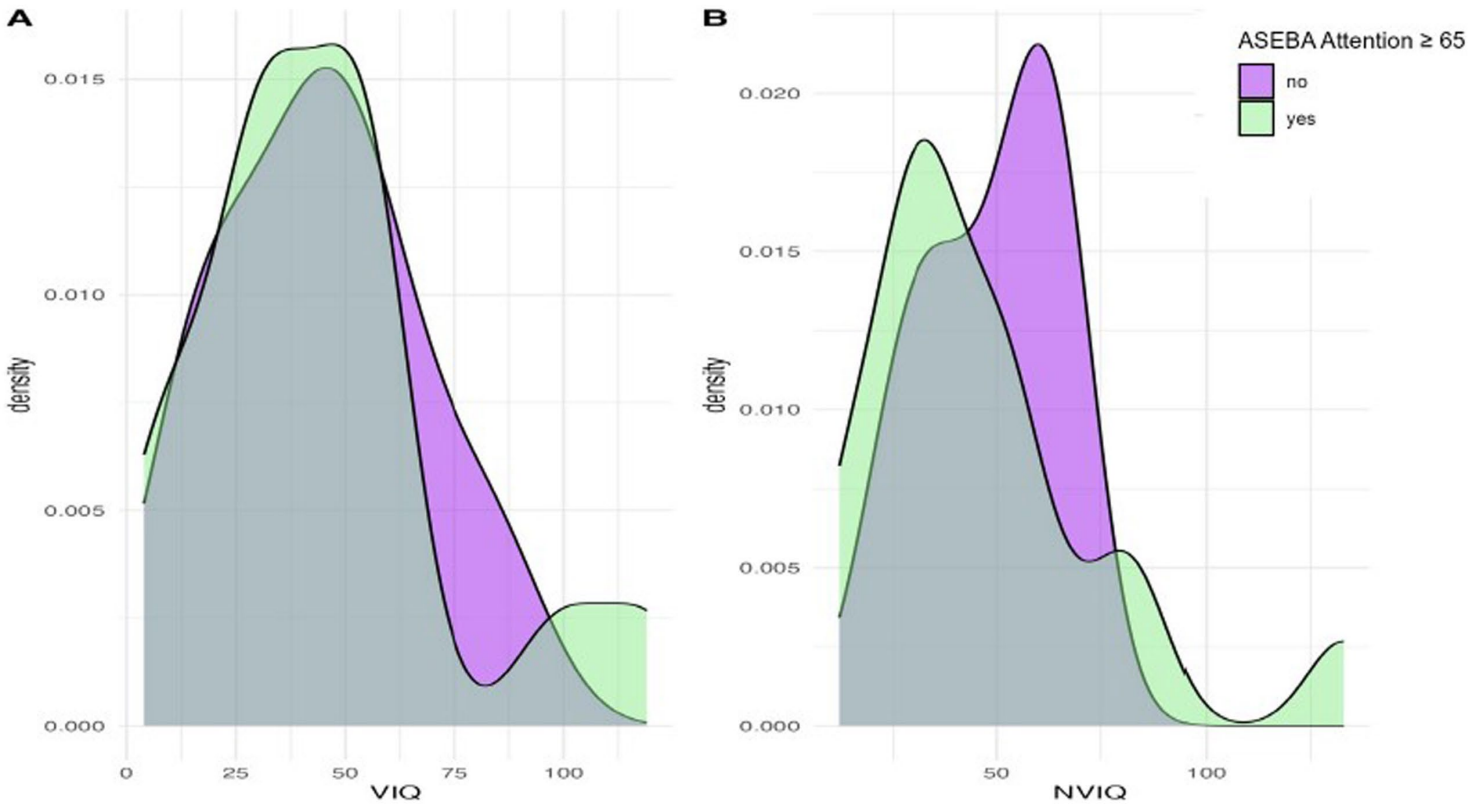
Density plots of T-scores on the ASEBA assessments, with a vertical line demarcating clinically significant scores (T-scores 65 or higher).

Descriptive statistics for scores on the BRIEF subscales, domain scores, and ASEBA subscales are presented in Table 3 and in Figures 2, 3. Descriptive statistics for the D-KEFS were omitted due to the low rates of completion.

On the BRIEF, the most reported executive functioning challenges were self-monitoring, working memory, and planning/organization, as shown in Figure 2. Means ranged across the subscales, from 46.9 (Emotional Control) up to 69.7 (Working Memory). When considering scores in relation to clinical significance, the percentage of the group exceeding clinical thresholds on BRIEF subscales also ranged substantially. Participants were least likely to fall above the clinical threshold on Emotional Control (10%) and Shift (20%), and substantially more likely to fall above the threshold on Working Memory (70%) and Self-Monitoring (66.7%). With regard to BRIEF Domain Scores, few participants exceeded the clinical threshold for the Behavioral Regulation Index (16.7%), whereas rates were higher for the Metacognition Index (60%).

On the ASEBA, participants commonly had elevated concerns on both the Attention and ADHD subscales, relative to chronological same-aged peers (Figure 3). On the ADHD subscale the group mean was slightly below the clinically significant threshold, with the mean for the Attention scale falling above the threshold. Most participants who scored in the clinically significant range on the ASEBA ADHD subscale were also reported to have clinically significant Attention difficulties (*n* = 13) and 9 individuals did not score as having clinically significant concerns on either subscale.

### 3.3 Executive functioning in relation to phenotypic features

Associations between EF and other phenotypic features were first examined using Spearman’s correlations, with a focus on ASEBA ADHD and Attention subscales as there were more complete data. Phenotypic features included mental and chronological age, IQ, and ASD features. Results are presented in Table 4. The ADHD subscale was not associated with age, IQ, or autism traits. Greater difficulties on the Attention subscale were significantly associated with lower nonverbal IQ and stronger autism features on the social awareness, social cognition, and social communication domains, as well as with stronger autism features overall.

**Table 4:** Spearman Correlations between ASEBA subscales and cognitive functioning and autism traits.

Variable	ASEBA ADHD	ASEBA attention
Nonverbal mental age (*n* = 22)	−0.23	−0.38
Verbal mental age (*n* = 22)	−0.30	−0.36
Chronological age (*n* = 27)	−0.06	−0.29
VIQ (*n* = 25)	−0.27	−0.34
NVIQ (*n* = 25)	−0.34	−0.42*
FSIQ (*n* = 26)	−0.27	−0.36
SRS awareness (*n* = 27)	0.34	0.48
SRS cognition (*n* = 27)	0.19	0.49**
SRS motivation (*n* = 27)	0.16	0.24
SRS communication (*n* = 27)	0.20	0.48*
SRS RRBs (*n* = 27)	0.29	0.38
SRS total (*n* = 27)	0.25	0.42*

*p < 0.05, **p < 0.01, ***p < 0.001. ASEBA, Achenbach System of Empirically Based Assessment; ADHD, Attention Deficit Hyperactivity Disorder; VIQ, Verbal IQ; NVIQ, Nonverbal IQ; FSIQ, Full Scale IQ; SRS, Social Responsiveness Scale; RRBs, Restricted, Repetitive Behaviors and Interests.

**Figure 4:**
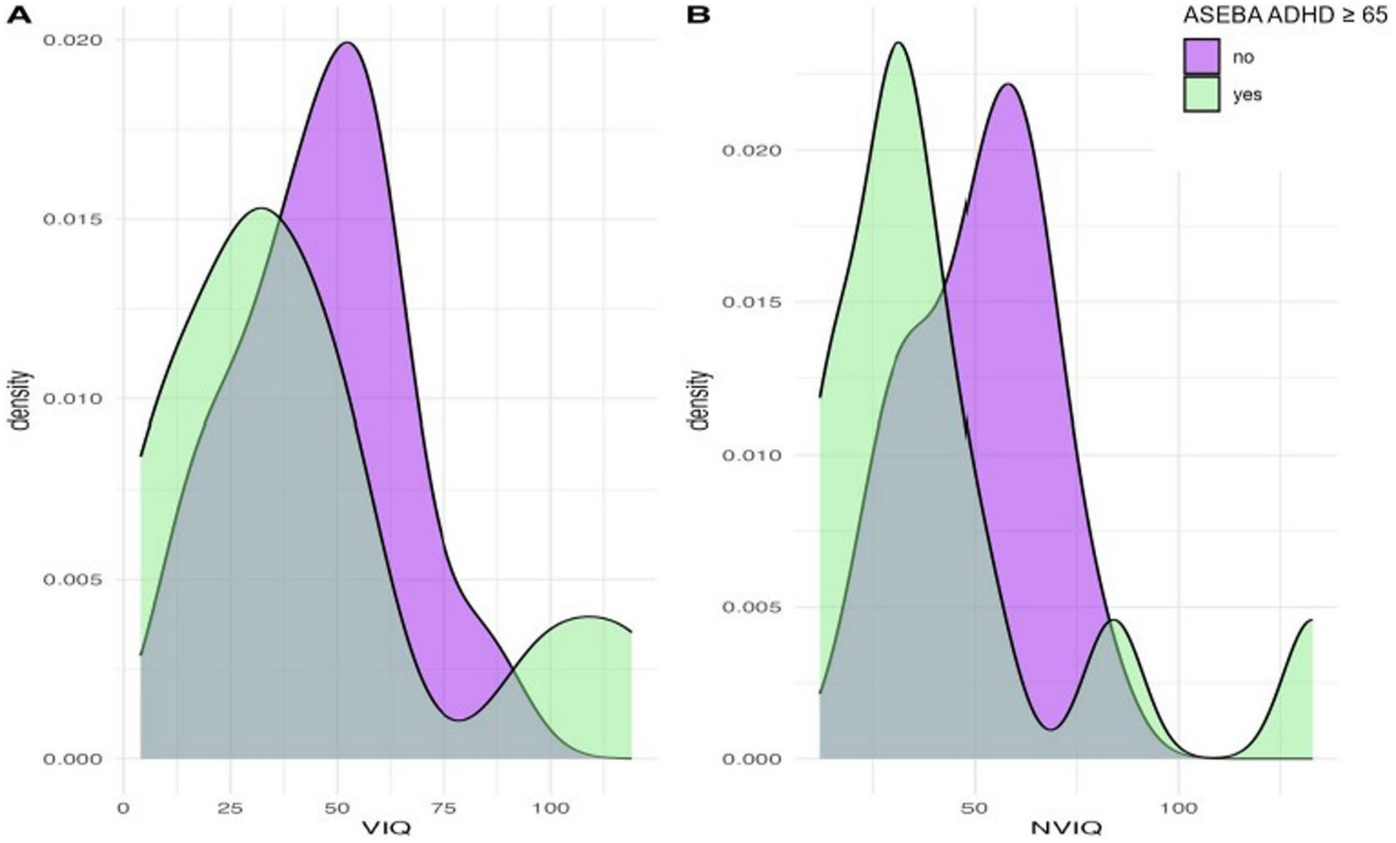
Density plots comparing IQ for those above and below the cut-point for clinically significant concerns on the ASEBA ADHD subscale.

**Figure 5:**
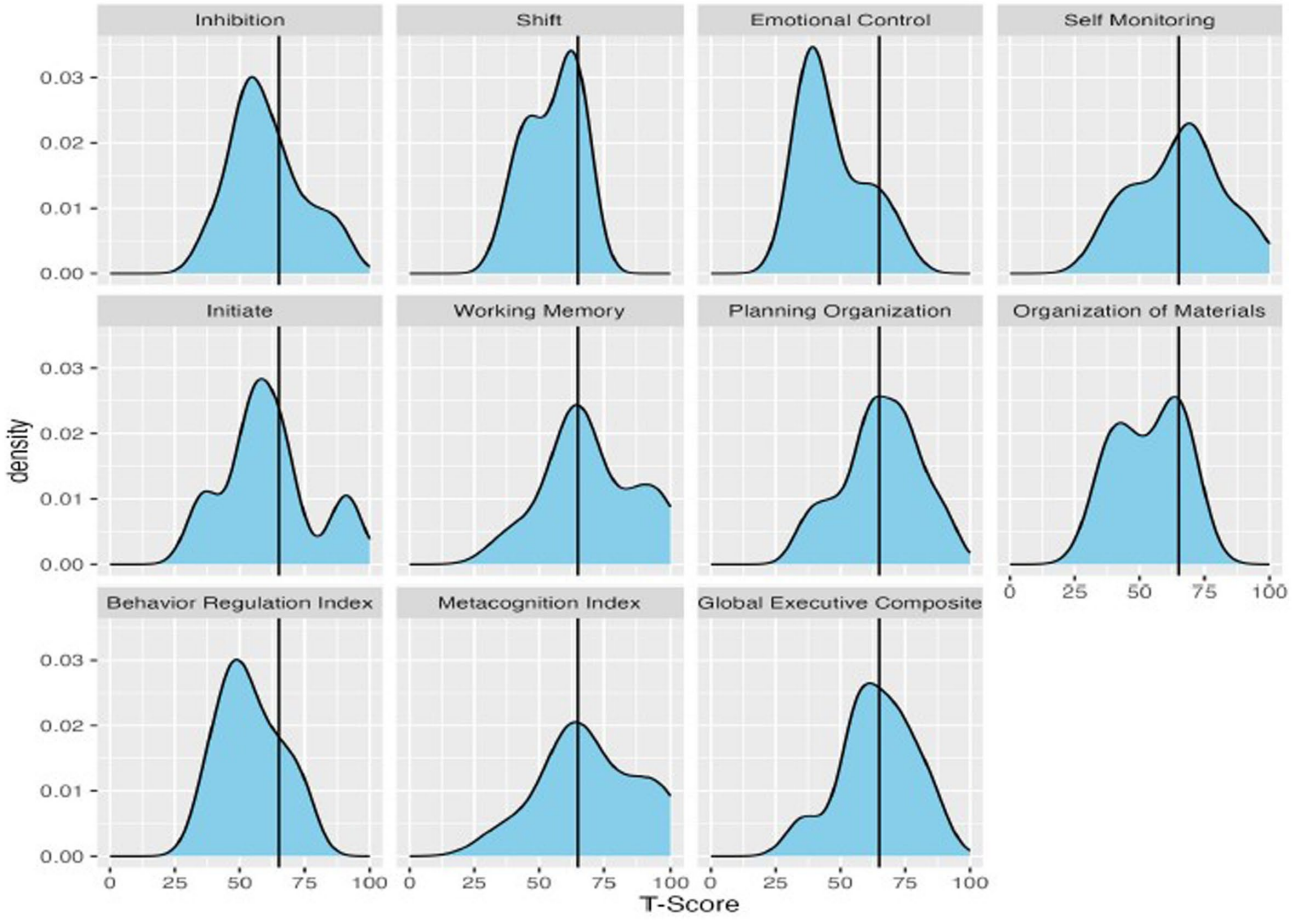
Density plots comparing IQ for those above and below the cut-point for clinically significant concerns on the ASEBA Attention subscale.

We next considered phenotypic features (VIQ, NVIQ, and autism traits) in relation to clinical significance on the ASEBA ADHD and Attention subscales. There was a significant difference in NVIQ between those above and below the clinical significance cut off on the ADHD subscale (*t (*22*)*=2.12, *p* = 0.045), such that individuals with scores in the clinically significant range on the ADHD subscale were more likely to have a lower NVIQ (Figure 4). There were no other significant findings, including no differences related to VIQ and ADHD (*p* = 0.285), NVIQ and Attention (*p* = 0.423), or VIQ and Attention (*p* = 0.646), as shown in Figures 4, 5. There was also not a significant difference in overall autism traits (SRS-2 total T-score) between those above or below the clinically significant threshold on the ADHD subscale (*p* = 0.459) or the Attention subscale (*p* = 0.488). There was not a significant difference in chronological age for those above or below the clinical significance cut off on the ADHD subscale (*p* = 0.335) or the Attention subscale (*p* = 0.360).

Finally, we examined the relation between caregiver-reported executive functioning and ADHD diagnosis in the sample. For the two participants within the group who had caregiver-reported existing ADHD diagnoses, the one participant with ADHD for whom the BRIEF was available scored below the clinically significant cut-point on the BRIEF global executive composite (i.e., overall executive functioning score; GEC). Conversely, 5 participants without caregiver-reported existing ADHD diagnosis scored above the clinical cut-point on the BRIEF GEC.

On the ASEBA, both participants with caregiver-reported existing ADHD diagnoses scored above the clinically significant cut-point on the ADHD and Attention Subscales. In addition, another 11 participants without caregiver-reported existing ADHD diagnoses also scored above the clinical cut-point on the ADHD subscale and an additional 15 participants without ADHD diagnoses scored above the clinical cut-point on the attention subscale.

## 4 Discussion

Our findings in this current, relatively large sample and large assessment battery were consistent with a prior behavioral characterization of ADHD and attention problems in *DYRK1A* syndrome (Fenster et al., 2022). Importantly, increasing calls to action suggest the need to develop and expand clinical measurements for rare genetic subgroups (Downs et al., 2024), in part due to the growing need for appropriate outcomes measures to be used in clinical trials (Hecker et al., 2024). Here, we assessed the current state of executive functioning assessments for individuals with *DYRK1A* syndrome as well as the relation among executive functioning challenges with other phenotypic features associated with *DYRK1A* syndrome.

### 4.1 Neurocognitive assessments

Executive functioning, an important domain of cognitive functioning, can be difficult to assess in individuals with multiple neurodevelopmental disorders. As questions remain about the most beneficial way to assess executive functioning using neurocognitive assessments for individuals with intellectual disability, and as neurocognitive assessments measure different aspects of executive functioning compared to caregiver report, it is important to discuss the feasibility of existing measures (Fidler and Lanfranchi, 2022). Considering *DYRK1A* syndrome specifically, the mean mental age range in our sample was 3 months to 8 years; however, most existing measures that are used in the general population extend only as early as school-age (Delis et al., 2001). Thus, there may be a need for measures for individuals with intellectual disability and/or that target a more inclusive developmental range. For individuals with *DYRK1A* syndrome with lower expressive verbal abilities, neurocognitive tasks may need to require minimal to no verbal production, have minimal verbal instructions, and have minimal motor demands due to the high rates of individuals being nonverbal and unable to point to communicate (van Bon et al., 2021). This would likely require the creation of novel executive functioning tasks that may provide objective measures, given that most existing neurocognitive assessments cannot readily dissociate core executive functioning difficulties from difficulties due to motor or language impairment.

### 4.2 Caregiver reports

On broad-based, standardized caregiver-report questionnaires, there was a range of presentations, with elevated challenges in self-monitoring, working memory, planning/organization, and overall executive functioning, on average. This pattern is largely consistent with areas of relative weakness that were identified in both preschoolers and school-aged youth with Down syndrome (Onnivello et al., 2022). Although approximately half the sample exhibited challenges in overall executive functioning, there were many executive functioning subdomains with relatively lower rates of impairment. Unlike other cognitive and neurodevelopmental skills in which impairment appears to be universal, there was broader range and marked variability across individuals within this group (Van Bon et al., 2016; Kurtz-Nelson et al., 2023; van Bon et al., 2011; Earl et al., 2017). Other research in intellectual disability indicates executive function is not a unitary construct, but rather group-level profiles show strengths and challenges in different executive functioning domains (Fidler and Lanfranchi, 2022). Syndrome-specific profiles have been identified (e.g., Down syndrome was characterized by challenges in shifting and working memory, with relative strengths in emotional control and organization), so the current investigation of group-level profiles of executive functioning in individuals with *DYRK1A* syndrome specifically yields a more nuanced understanding of *DYRK1A* syndrome (Spaniol and Danielsson, 2022; Fidler and Lanfranchi, 2022; Onnivello et al., 2022). However, on average, executive functioning skills appear to be impacted in this population. These results highlight the importance of continuing deep phenotyping of ASD-associated genetic disorders, such as *DYRK1A*.

Although executive functioning skills underlie ADHD, there was a low prevalence of existing ADHD diagnoses for families in this study (Antshel et al., 2014). Clinicians and medical providers may be less likely to consider ADHD diagnoses for this population in the context of existing autism and intellectual disability diagnoses, particularly given that autism, intellectual disability, and ADHD diagnoses can include some behavioral or symptom overlap (Mason and Scior, 2004). The phenomenon of diagnostic overshadowing, or the tendency to attribute all behaviors to intellectual disability (Mason and Scior, 2004; Hendriksen et al., 2015) occurs in other populations with neurological disabilities and can result in under-recognition of co-occurring mental health concerns. Further complicating diagnostic decisions, there was little agreement between the two broad-based measures of executive functioning, as individuals were inconsistently elevated on the ASEBA and BRIEF. Based on our results, the ASEBA more closely aligned with clinician perspectives, compared to the BRIEF, as there was more agreement between the ASEBA and existing diagnoses. However, clinicians and providers may need to consider the limitations of measures that were not normed on samples with intellectual disability.

### 4.3 Executive functioning assessments

Given discrepancy between chronological age and mental age in *DYRK1A* syndrome and other genetic-NDD syndromes, identifying measures that can adequately assess executive functioning without infantilizing the individual are critically needed. Caregiver report and neurocognitive assessments have been found to measure different aspects of executive functioning (Toplak et al., 2013), and as such, both classes of assessments are valuable in identifying phenotypic features in a new population. One helpful advancement may be the extension of established tools to encompass a broader developmental range, which will allow for more options in test selection and the potential for adaptation into this population. For instance, the NIH Infant and Toddler Toolbox (which is anticipated to contain executive functioning modules) is expected to be released in late 2024, which may prove useful for individuals with *DYRK1A* syndrome (Gershon et al., 2024). The ASEBA and BRIEF have been shown to be reliable and valid in other populations of youth with intellectual disability (e.g., youth with Down syndrome), and a comparison of the BRIEF-P and BRIEF-2 in youth with Down syndrome indicated that the form administered should match the child’s chronological age rather than mental age (Esbensen et al., 2018; Esbensen et al., 2024). Nonetheless, clinicians working with individuals with *DYRK1A* syndrome will need to consider the validity of these forms with that population specifically, particularly in instances of differences between chronological and mental age.

This study is not without limitations. Although genetics-first approaches allow for a broader range of phenotypes to be included, access to genetic testing for children with NDD in clinical practice is still inconsistent and limited (Savatt and Myers, 2021; van der Sanden et al., 2023; Zhang et al., 2024); thus our sample likely does not reflect the true heterogeneity of profiles in this condition. Due to the rarity of *DYRK1A* syndrome, the sample size is small and primarily consisted of youth and adolescents. Following participants as they age will be important for understanding the trajectory of executive functioning in those with *DYRK1A* syndrome. Longitudinal follow up may be especially relevant to understanding executive functioning as educational and behavioral expectations increase with age. Finally, given the range of variant types reported in this study, variant-level correlations are challenging.

In sum, the current study demonstrated a need for more objective, behaviorally appropriate measures of executive functioning for individuals with *DYRK1A* syndrome and intellectual disability broadly. Contributing to our understanding of the phenotype associated with *DYRK1A*, there appears to be a range of executive functioning skills, although many caregivers do report clinically significant challenges in executive functioning that warrant attention, even when overlapping with other diagnostic profiles. Future research may consider investigating the impact of ADHD behavioral and/or medical interventions to address executive functioning concerns in this population.

## Data Availability

The datasets presented in this study can be found in online repositories. The names of the repository/repositories and accession number(s) can be found at: https://nda.nih.gov/, 2093.
